# Intraosseous inflammatory myofibroblastic tumor of the mandible with a novel *ATIC-ALK* fusion mutation: a case report

**DOI:** 10.1186/s13000-016-0586-z

**Published:** 2016-11-15

**Authors:** Yoko Tateishi, Koji Okudela, Shigeo Kawai, Takehisa Suzuki, Shigeaki Umeda, Mai Matsumura, Mitomu Kioi, Kenichi Ohashi

**Affiliations:** 1 Department of Pathology, Yokohama City University Graduate School of Medicine, Yokohama, Japan; 2 Department of Pathology, Japanese Red-Cross Musashino Hospital, Tokyo, Japan; 3 Department of Oral and Maxillofacial Surgery, Yokohama City University Graduate School of Medicine, Yokohama, Japan

**Keywords:** Inflammatory myofibroblastic tumor, Mandible, Anaplastic lymphoma kinase gene, *ATIC-ALK* fusion, Fluorescence in situ hybridization, 5′ rapid amplification of cDNA ends, RT-PCR

## Abstract

**Background:**

Inflammatory myofibroblastic tumor (IMT) is a rare low-grade malignant neoplasm with a predilection for children and young adults, and typically arises in the lung, abdominopelvic region, and retroperitoneum. IMTs in the maxillofacial region are extreme rare. Approximately 50% of IMT harbor rearrangements of the anaplastic lymphoma kinase (*ALK*) gene at 2p23 with various fusion partners.

**Case presentation:**

We herein report a case of intraosseous IMT of the mandible with a novel *ATIC-ALK* fusion. Tooth 43 did not erupt after the loss of tooth 83 in an 11-year-old girl with no previous history of trauma. Panoramic tomography showed a unilocular radiolucent lesion in the right anterior mandible resorbing the root of tooth 42 and the medial side of the root of tooth 44. Computed tomography revealed a well- circumscribed 3-cm osteolytic lesion of the right anterior mandible eroding the buccal cortical plate. The entire lesion was curetted out. A histopathological examination revealed the proliferation of plump spindle cells with a storiform architecture and lymphocytes scattered around spindle cells. The spindle cells showed diffuse cytoplasmic staining for ALK by immunohistochemistry. A fluorescence *in situ* hybridization analysis revealed the translocation of a part of the *ALK* gene locus at chromosome 2p23. A rapid amplification of cDNA ends analysis confirmed the rearrangement of *ALK* and identified *ATIC* as a partner of this *ALK* fusion mutant.

**Conclusion:**

To the best of our knowledge, this is the first case of intraosseous IMT of the mandible with a novel *ATIC-ALK* fusion. We also herein reviewed similar tumors reported in the literature.

## Background

Inflammatory myofibroblastic tumors (IMTs) are more frequent in children and young adults, and typically arise in the lung, abdominopelvic region, and retroperitoneum [1, 2]. Unusual sites of involvement include the head and neck, genitourinary tract, heart, extremities, and central nervous system [2]. While these tumors are rare in the head and neck, they are even more uncommon in the mandible [3].

IMTs are mesenchymal neoplasms of intermediate malignant potential and histologically characterized by the proliferation of fibroblasts and myofibroblasts admixed with lymphocytes, plasma cells, eosinophils, and histiocytes [1]. The histological features of IMTs are often diagnostic, particularly through anaplastic lymphoma kinase (ALK) staining. The *ALK* gene has been implicated in the pathogenesis of IMTs, which supports the neoplastic origin of these tumors. Approximately 50% of IMTs harbor rearrangements in the *ALK* gene at 2p23 [4]. However, to the best of our knowledge, an *ALK* fusion mutation has not yet been demonstrated in IMTs in the jawbone.

We herein report a case of an intraosseous IMT in the mandible including a novel *ATIC-ALK* fusion mutation.

## Case presentation

### Clinical features

**Fig. 1:**
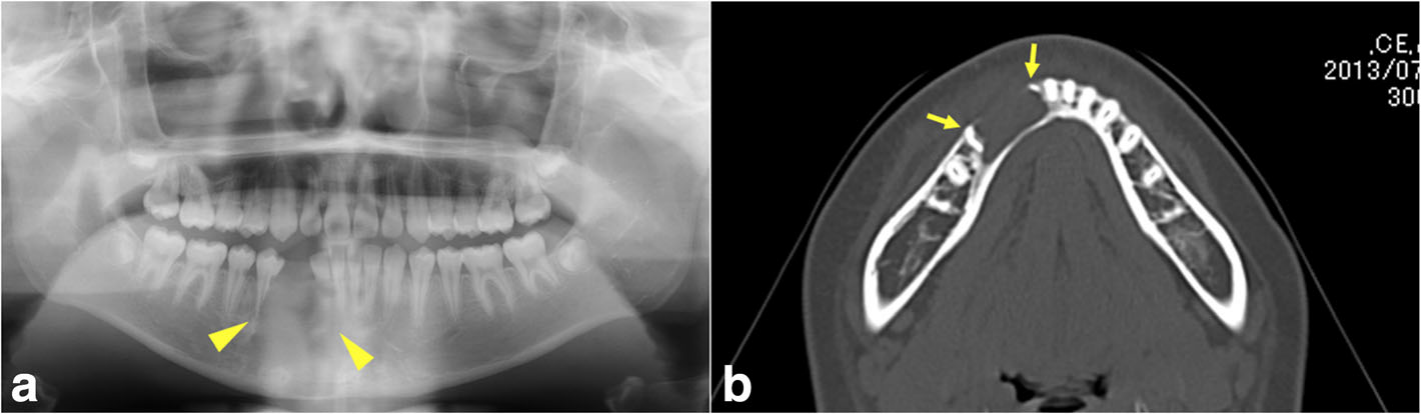
**a** Panoramic tomography showed the resorption of the root of tooth 42 and medial side of the root of tooth 44 (*arrow heads*). **b** Computed tomography revealed a well- circumscribed 3-cm osteolytic lesion in the right mandible that eroded the buccal cortical plate and resorbed the roots of tooth 42 and tooth 44 (*arrows*)

An 11-year-old girl presented with the delayed eruption of tooth 43 (two digit tooth notation system of the FDI) and mental nerve palsy. She had no history of trauma. An intraoral examination revealed that tooth 43 had not erupted after the loss of tooth 83 and the gingiva and alveolar mucosa of the right mandible were normal. Panoramic tomography showed a unilocular radiolucent lesion in the right anterior mandible resorbing the root of tooth 42 and the medial side of the root of tooth 44 (Fig. 1a). Tooth 43 was not impacted in the mandibular bone. Computed tomography showed a well-circumscribed non-expansile 3-cm osteolytic lesion in the right anterior mandible that eroded the buccal cortical plate and resorbed the root of tooth 42 and the medial side of the root of tooth 44 (Fig. 1b). Root resorption and cortical plate erosion suggested a locally aggressive tumor. The tumor lesion was entirely curetted out. No recurrence was detected in 18 months of follow-up.

### Histopathological features and differential diagnosis

**Fig. 2:**
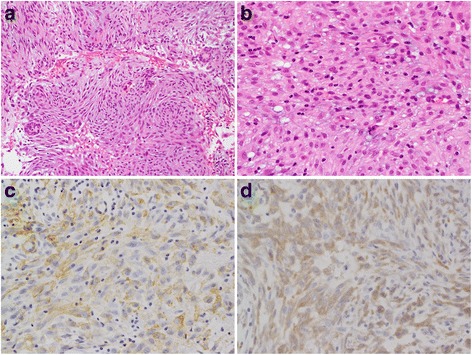
Hematoxylin and eosin staining (**a**, **b**) and immunohistochemical staining (**c**, **d**). **a** The proliferation of plump spindle cells with a storiform architecture with scattered lymphocytes. Epithelioid components with an appearance suggestive of odontogenic epithelioid cells were detected. **b** Spindle cells contained ovoid nuclei with a pale eosinophilic cytoplasm. **c** Spindle cells were positive for SMA. **d** Spindle cells were positive for ALK

A histopathological investigation revealed plump spindle cell proliferation with a storiform architecture and lymphocytes scattered around the spindle cells (Fig. 2a, b). The background stroma included a focal myxoid matrix. These spindle cells had ovoid nuclei with a pale eosinophilic cytoplasm, suggesting a myofibroblastic, myoepithelial, or fibroblastic lineage. Small islands of an odontogenic epithelium were detected. No mitosis or necrosis was observed. The histomorphological findings suggested that spindle cells were of a myofibroblastic, myoepithelial, or fibroblastic lineage. Small islands of an odontogenic epithelium suggested that this tumor was an odontogenic tumor. High-grade malignant tumors were excluded because neither mitosis nor necrosis was observed. Differential diagnoses included myofibroma, myoepithelioma, an odontogenic tumor, solitary fibrous tumor, giant cell granuloma, and IMT.

### Immunohistochemical findings

Immunohistochemical staining revealed that spindle cells were positive for α-smooth muscle actin (SMA) (Fig. 2c), ALK (Fig. 2d), CD10, and glial fibrillary acidic protein (GFAP), and negative for cytokeratin AE1/AE3, S100 proteins, and p63. The Ki67 labeling index was 3%.

### Molecular pathological findings

#### FISH

**Fig. 3:**
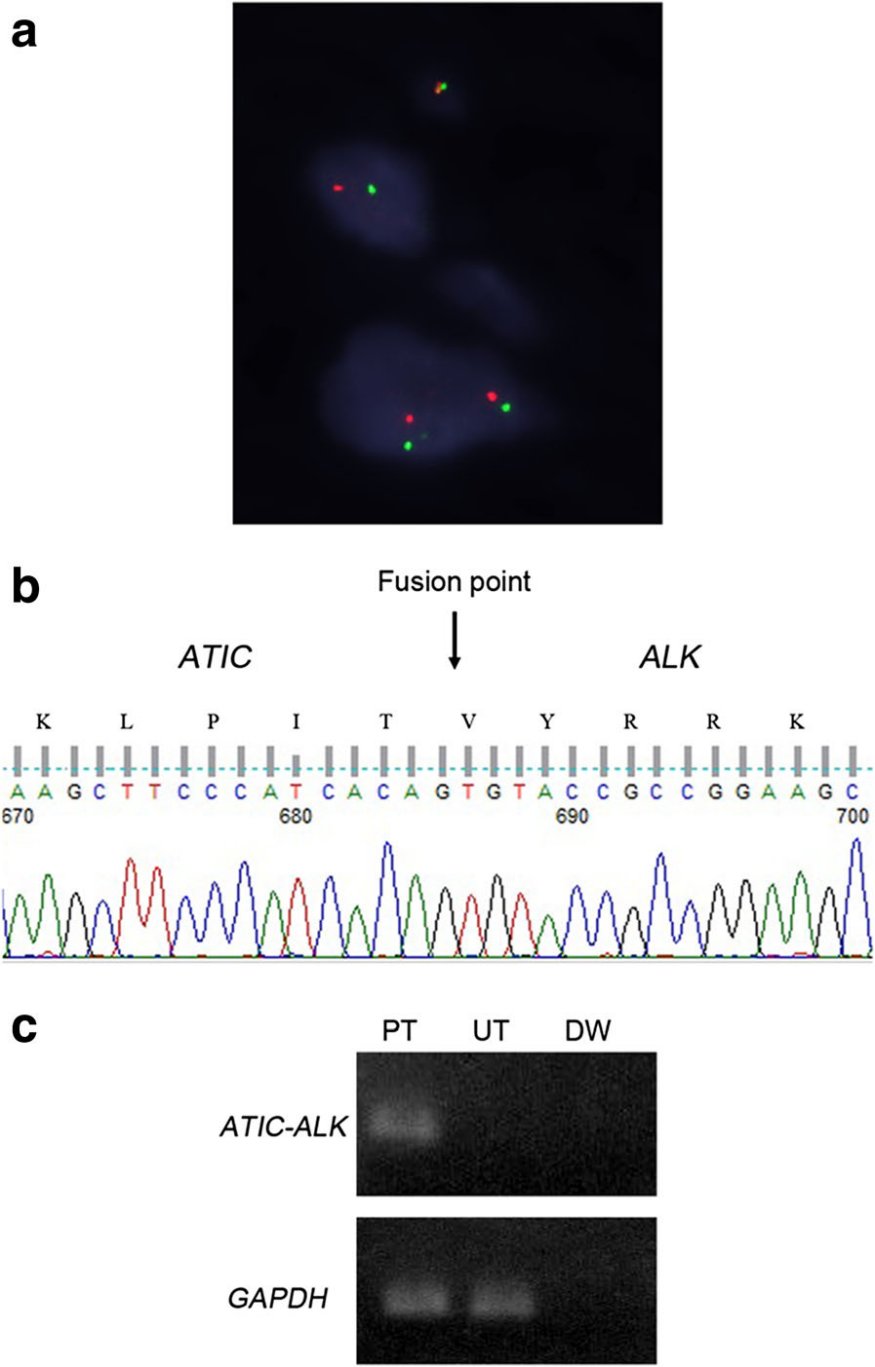
**a** FISH assay with a break apart probe for the *ALK* gene shows one intact yellow signal and one separated red and green signal per nucleus in tumor cells, indicating the presence of a rearrangement in the *ALK* gene. **b** The 5′ RACE analysis identified *ATIC* as a fusion partner. The sequence of the second round PCR product covering the fusion point (arrow) is shown. **c** The results of the RT-PCR analysis for *ATIC-ALK* are shown. PT: Present tumor, UT: Unrelated tumor (lung tumor), DW: Distilled Water, GAPDH: glyceraldehydes-3-phosphate dehydrogenase

A fluorescence *in situ* hybridization (FISH) *(ALK)* analysis was performed on a formalin-fixed paraffin-embedded (FFPE) tissue block. Rearrangements in the *ALK* gene at chromosome band 2p23 were detected by FISH utilizing a Vysis ALK break apart probe (Abott Molecular). The FISH analysis revealed the translocation of a part of the *ALK* gene locus (Fig. 3a).

#### RACE

Total RNA was extracted from frozen tissue using an RNeasy mini kit (Qiagen) according to the manufacturer’s instructions. In order to obtain cDNA fragments corresponding to a novel *ALK* fusion gene, a rapid amplification of cDNA ends (RACE) analysis was performed using the 5′-full RACE core set (TaKaRa Bio) according to the manufacturer’s instructions. First-strand cDNA synthesis was performed with a 5′ end-phosphorylated RT primer (5′-ATCTGGGCCTTGTATTTATCACTC). The primer set used for the first round of PCR was the A1 primer (5′-ACTTCCTGGTTGCTTTTGCTGGGGTAT) [5] and S1 primer (5′-AGAAGGAGCCACACGACAGGGGTAA). The primer set used for the second round of PCR was the S2 primer (5′-CTCCTTCACAAACCAGAGACCAA) and A2 primer (5′-TTCAGGCAGCGTCTTCACAG). The second round PCR product covering the break point was sub-cloned into a pT7Blue plasmid vector (TaKaRa Bio). The DNA sequence was analyzed using universal primers (M13M4 primer or T7 promoter primer) and the Big-dye terminator ver3.1 kit (Applied Biosystems). A RACE analysis confirmed a rearrangement in ALK and identified ATIC (NM_004044) as a partner of this ALK fusion mutant (Fig. 3b).

#### RT-PCR

First-strand cDNA was synthesized from total RNA using the SuperScript First-Strand Synthesis System (Invitrogen) according to the manufacturer’s instructions. RT-PCR was used to confirm the presence of the *AITC-ALK* fusion gene in this case. The primer set used was as follows: forward (5′-CCAAGCTTCCCATCACAGTGTACC) and reverse (5′-GAGGTCTTGCCAGCAAAGCAGTAG). RT-PCR was used to confirm the presence of glyceraldehyde-3-phosphate dehydrogenase (GAPDH) in this case. The primer set used was as follows: forward (5′-TGGAGTCCACTGGCGTCTTC) and reverse (5′-ATGACGAACATGGGGGCATCAG). A RT-PCR analysis confirmed the *ATIC-ALK* fusion in tumor cells (Fig. 3c).

Based on these results, the final diagnosis was IMT.

## Discussion

We herein presented the first case of IMT with an *ATIC-ALK* fusion mutation primarily in the maxillofacial region. IMTs are rare mesenchymal tumors. Different terms have been applied to these lesions, namely, xanthogranuloma, plasma cell granuloma, inflammatory pseudotumor (IPT), and inflammatory myofibroblastic sarcoma [1, 6, 7]. The diverse nomenclature is descriptive and reflects the uncertainty regarding the true biological nature of these lesions. In 2013, the World Health Organization classification of soft tissue and bone defined IMT as a distinctive neoplasm composed of myofibroblastic and fibroblastic spindle cells accompanied by an inflammatory infiltrate of plasma cells, lymphocytes, and/or eosinophils [1]. The expression of ALK proteins is detected by anti-ALK immunohistochemistry in approximately 50% of IMT cases. ALK protein expression has been shown to reliably correlate with *ALK* gene rearrangements [8]. These lines of evidence support ALK-positive IMT being a distinct neoplastic entity. ALK immunostaining is an aid in the pathological diagnosis of IMTs, as well as in the differentiation of IMTs from other spindle cell neoplasms that fall within the broad category of IPTs. We reviewed 12 cases of intraosseous tumors of the mandible with similar histological features in the literature (Table 1) [7, 9–19]. Six cases were diagnosed as IPTs and 6 as IMTs in the mandible. ALK-positive IMT/IPT in the mandible was initially reported in 2005 [10] and only 4 cases including ours have been reported since then [10, 11, 19].

**Table 1:** Clinicopathological features of IMTs in the mandible described in the present and previous reports

No.	Authors (yr.)	Age (yr.)	Sex	Diagnosis	ALK-IHC	FISH	RT-PCR
1	Zegarelli (1974) [7]	56	F	Granuloma (?IPT)	NA	NA	NA
2	Inui (1993) [9]	63	M	IPT	NA	NA	NA
3	Brooks (2005) [10]	82	F	IMT	positive	NA	NA
4	Poh (2005) [11]	42	F	IMT	positive	NA	NA
5	Oh (2008) [12]	20	F	IPT	NA	NA	NA
6	Johann (2008) [13]	33	M	IPT	NA	NA	NA
7	Satomi (2010) [14]	14	F	IMT	negative	NA	NA
8	Date (2010) [15]	70	M	IPT	negative	NA	NA
9	Sasagawa (2011) [16]	60	M	IMT	NA	NA	NA
10	Gawande (2012) [17]	20	M	IPT	NA	NA	NA
11	Sah (2013) [18]	30	M	IMT	negative	NA	NA
12	Stringer (2014) [19]	16	M	IMT	positive	NA	NA
13	Present case	11	F	IMT	positive	positive	*ATIC-ALK*

*ALK* anaplastic lymphoma kinase, IHC immunohistochemistry, FISH Fluorescene in situ hybridization, F female, M male, IPT Inflammatory pseudotumor, IMT Inflammatory myofibroblastic tumor, NA data not available

Thus, IMT may very rarely occur in the mandible. Otherwise, IMT may often be misdiagnosed because difficulties are associated with its histological differentiation from other spindle cell neoplasms and reactive lesions. Differential diagnoses include posttraumatic spindle cell nodules, giant cell granuloma, myofibroma, myoepithelioma, ameloblastic fibroma, odontogenic myxofibroma, IMT, a solitary fibrous tumor, low-grade myofibroblastic sarcoma, myofibrosarcoma, leiomyosarcoma, and spindle cell carcinoma. The immunohistochemical expression of the ALK protein is a very important hallmark for differentiating IMT from those described above. Therefore, if spindle cell lesions are detected in the jawbone, IMT must be included in the differential diagnosis and an immunohistochemical examination of ALK protein expression is needed.

**Fig. 4:**
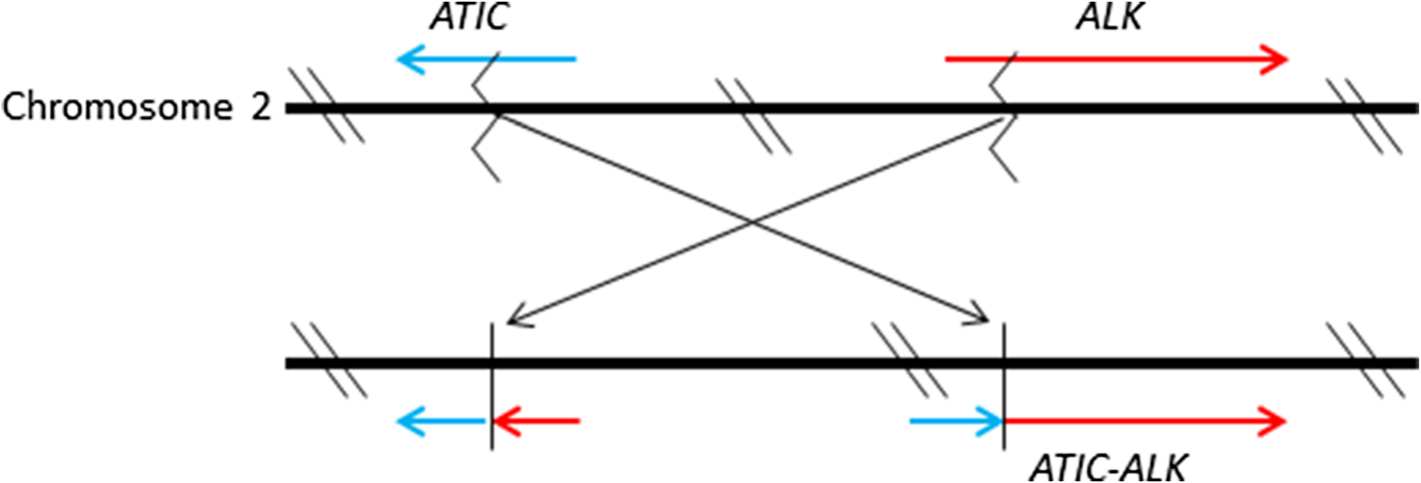
The hypothetical schema of the gene fusion between *ALK* and *ATIC. ATIC*-*ALK* fusion is generated by the pericentric inversion of chromosome 2 at the breakpoints of p23 and q35 [inv(2)(p23q35)], in which the *ALK* and *ATIC* genes are localized

The present tumor was confirmed to have the *ATIC-ALK* fusion mutation. Different *ALK* fusion partners, including *TPM3*, *TPM4*, *CLTC*, *CARS*, *RANBP2*, *ATIC*, *SEC31L1*, *PPFIBP1*, and *DCTN1*, in which *TPM3* is the most common partner, have been identified in IMTs [5, 20–26]. *ATIC-ALK* fusion mutations are very rare, comprising approximately 1% of ALK-fusion mutations in anaplastic large cell lymphomas (ALCL) [27]. The hypothetical schema of the gene fusion between *ATIC* and *ALK* is shown in Fig. 4 [28]. To the best of our knowledge, one case of urinary bladder IMT with the *ATIC-ALK* fusion mutation and one case of IMT arising from the hyoid bone in a neonate with the *ATIC-ALK* fusion have been reported in the literature [23, 29]. Thus, the present case is the first IMT with the *ATIC-ALK* fusion mutation primarily in the mandible.

The management of IMTs needs to entail complete surgical resection, including postoperative reassessments for at least 10 years [30]. Although the number of IMTs in the jawbone is limited, they appear to exhibit a favorable clinical course. However, it is important to emphasize that IMTs occasionally behave in an aggressive manner. Poh et al. reported the local recurrence of an intraosseous mandibular IMT 14 months after initial surgery [11]. Sasagawa et al. described a case of multiple IMTs involving the cranium, mandible, ischium, and calcaneus [16]. The ALK inhibitor, crizotinib, was recently demonstrated to be clinically beneficial for IMTs with ALK fusion mutations [26, 31–33].

## Conclusion

IMTs are rare lesions in the mandible. The general consensus is that IMTs encompass a group of heterogeneous lesions with various biological behaviors. However, a proportion of cases are genuine neoplasms with the involvement of *ALK* fusion mutations. The recognition of this diagnostic entity is important for selecting appropriate treatments.

To the best of our knowledge, we are the first to describe IMT with the *ATIC-ALK* fusion mutation in the mandible.

## Abbreviations

IMT: Inflammatory myofibroblatic tumor
ALK: Anaplastic lymphoma kinase
FISH: Fluorescence *in situ* hybridization
RACE: Rapid amplification of cDNA ends
RT-PCR: Reverse transcription polymerase Chain reaction
IPT: Inflammatory pseudotumor

## References

[1] Coffin CM, Fletcher JA. Inflammatory myofibroblastic tumour. In Fletcher CDM, Bridge JA, Hogendoorn PCW, Mertens F eds. World Health Organization classification of tumours soft tissue andbone. Lyon: IARC Press; 2013. p. 83–4.

[2] Coffin CM, Hornick JL, Fletcher CD (2007). Inflammatory myofibroblastic tumor: comparison of clinicopathologic, histologic, and immunohistochemical features including ALK expression in atypical and aggressive cases. Am J Surg Pathol.

[3] Park SB, Lee JH, Weon YC (2009). Imaging findings of head and neck inflammatory pseudotumor. AJR Am J Roentgenol.

[4] Cessna MH, Zhou H, Sanger WG, Perkins SL, Tripp S, Pickering D (2002). Expression of ALK1 and p80 in inflammatory myofibroblastic tumor and its mesenchymal mimics: a study of 135 cases. Mod Pathol.

[5] Lawrence B, Perez-Atayde A, Hibbard MK, Rubin BP, Dal Cin P, Pinkus JL (2000). TPM3-ALK and TPM4-ALK oncogenes in inflammatory myofibroblastic tumors. Am J Pathol.

[6] Rudy HN, Scheingold SS (1964). Solitary xanthogranuloma of the mandible; report of a case. Oral Surg Oral Med Oral Pathol.

[7] Zegarelli DJ, Rankow RM, Zegarelli EV (1974). A large dental granuloma (? inflammatory pseudotumor) with unusual features: report of case. J Am Dent Assoc.

[8] Cook JR, Dehner LP, Collins MH, Ma Z, Morris SW, Coffin CM (2001). Anaplastic lymphoma kinase (ALK) expression in the inflammatory myofibroblastic tumor: a comparative immunohistochemical study. Am J Surg Pathol.

[9] Inui M, Tagawa T, Mori A, Yoneda J, Nomura J, Fukumori T (1993). Inflammatory pseudotumor in the submandibular region. Clinicopathologic study and review of the literature. Oral Surg Oral Med Oral Pathol.

[10] Brooks JK, Nikitakis NG, Frankel BF, Papadimitriou JC, Sauk JJ (2005). Oral inflammatory myofibroblastic tumor demonstrating ALK, p53, MDM2, CDK4, pRb, and Ki-67 immunoreactivity in an elderly patient. Oral Surg Oral Med Oral Pathol Oral Radiol Endod.

[11] Poh CF, Priddy RW, Dahlman DM (2005). Intramandibular inflammatory myofibroblastic tumor--a true neoplasm or reactive lesion?. Oral Surg Oral Med Oral Pathol Oral Radiol Endod.

[12] Oh JH, Yim JH, Yoon BW, Choi BJ, Lee DW, Kwon YD (2008). Inflammatory pseudotumor in the mandible. J Craniofac Surg.

[13] Johann AC, Caldeira PC, Abdo EN, Sousa SO, Aguiar MC, Mesquita RA (2008). Inflammatory myofibroblastic tumor of the alveolar mucosa of the mandible. Minerva Stomatol.

[14] Satomi T, Watanabe M, Matsubayashi J, Nagao T, Chiba H (2010). A successfully treated inflammatory myofibroblastic tumor of the mandible with long-term follow-up and review of the literature. Med Mol Morphol.

[15] Date A, Yamagata K, Onizawa K, Yanagawa T, Karube R, Ishibashi N (2012). Inflammatory pseudotumor: report of a case in the mandible. Oral Maxillofac Surg.

[16] Sasagawa Y, Akai T, Itou S, Iizuka H (2011). Multiple intraosseous inflammatory myofibroblastic tumors presenting with an aggressive clinical course: case report. Neurosurgery.

[17] Gawande PD, Sambhus M, Garde JB, Halli R, Deshmukh V, Kulkarni A (2012). Aggressive inflammatory pseudotumor of the mandible. J Craniofac Surg.

[18] Sah P, Byatnal AA, Rao L, Narayanaswamy V, Radhakrishnan R (2013). Inflammatory myofibroblastic tumor: a rapidly growing soft tissue mass in the posterior mandible. Head Neck Pathol.

[19] Stringer DE, Allen CN, Nguyen K, Tandon R (2014). Intraosseous inflammatory myofibroblastic tumor in the mandible: a rare pathologic case report. Case Rep Surg.

[20] Bridge JA, Kanamori M, Ma Z, Pickering D, Hill DA, Lydiatt W (2001). Fusion of the ALK gene to the clathrin heavy chain gene, CLTC, in inflammatory myofibroblastic tumor. Am J Pathol.

[21] Cools J, Wlodarska I, Somers R, Mentens N, Pedeutour F, Maes B (2002). Identification of novel fusion partners of ALK, the anaplastic lymphoma kinase, in anaplastic large-cell lymphoma and inflammatory myofibroblastic tumor. Genes Chromosomes Cancer.

[22] Ma Z, Hill DA, Collins MH, Morris SW, Sumegi J, Zhou M (2003). Fusion of ALK to the ran-binding protein 2 (RANBP2) gene in inflammatory myofibroblastic tumor. Genes Chromosomes Cancer.

[23] Debiec-Rychter M, Marynen P, Hagemeijer A, Pauwels P (2003). ALK-ATIC fusion in urinary bladder inflammatory myofibroblastic tumor. Genes Chromosomes Cancer.

[24] Panagopoulos I, Nilsson T, Domanski HA, Isaksson M, Lindblom P, Mertens F (2006). Fusion of the SEC31L1 and ALK genes in an inflammatory myofibroblastic tumor. Int J Cancer.

[25] Takeuchi K, Soda M, Togashi Y, Sugawara E, Hatano S, Asaka R (2011). Pulmonary inflammatory myofibroblastic tumor expressing a novel fusion, PPFIBP1-ALK: reappraisal of anti-ALK immunohistochemistry as a tool for novel ALK fusion identification. Clin Cancer Res.

[26] Subbiah V, McMahon C, Patel S, Zinner R, Silva EG, Elvin JA (2015). STUMP un“stumped”: anti-tumor response to anaplastic lymphoma kinase (ALK) inhibitor based targeted therapy in uterine inflammatory myofibroblastic tumor with myxoid features harboring DCTN1-ALK fusion. J Hematol Oncol.

[27] Colleoni GW, Bridge JA, Garicochea B, Liu J, Filippa DA, Ladanyi M (2000). ATIC-ALK: a novel variant ALK gene fusion in anaplastic large cell lymphoma resulting from the recurrent cryptic chromosomal inversion, inv(2)(p23q35). Am J Pathol.

[28] Wlodarska I, De Wolf-Peeters C, Falini B, Verhoef G, Morris SW, Hagemeijer A (1998). The cryptic inv(2)(p23q35) defines a new molecular genetic subtype of ALK-positive anaplastic large-cell lymphoma. Blood.

[29] Owusu-Brackett N, Johanson R, Schindel DT, Koduru P, Cope-Yokoyama S (2013). A novel ALK rearrangement in an inflammatory myofibroblastic tumor in a neonate. Cancer Genet.

[30] Meis-Kindblom JM, Kjellstrom C, Kindblom LG (1998). Inflammatory fibrosarcoma: update, reappraisal, and perspective on its place in the spectrum of inflammatory myofibroblastic tumors. Semin Diagn Pathol.

[31] Butrynski JE, D’Adamo DR, Hornick JL, Dal Cin P, Antonescu CR, Jhanwar SC (2010). Crizotinib in ALK-rearranged inflammatory myofibroblastic tumor. N Eng J Med.

[32] Nishio M, Murakami H, Horiike A, Takahashi T, Hirai F, Suenaga N (2015). Phase I study of ceritinib (LDK378) in Japanese patients with advanced, anaplastic lymphoma kinase-rearranged non-small-cell lung cancer or other tumors. J Thorac Oncol.

[33] Jacob SV, Reith JD, Kojima AY, Williams WD, Liu C, Vila DL (2014). An unusual case of systemic inflammatory myofibroblastic tumor with successful treatment with ALK-inhibitor. Case Rep Pathol.

